# ATHB2 is a negative regulator of germination in *Arabidopsis thaliana* seeds

**DOI:** 10.1038/s41598-021-88874-5

**Published:** 2021-05-06

**Authors:** Rocío Soledad Tognacca, Monica Carabelli, Giorgio Morelli, Ida Ruberti, Javier Francisco Botto

**Affiliations:** 1 Consejo Nacional de Investigaciones Científicas y Técnicas, Instituto de Investigaciones Fisiológicas y Ecológicas Vinculadas a la Agricultura (IFEVA), Facultad de Agronomía, Universidad de Buenos Aires, Av. San Martín 4453, C1417DSE Buenos Aires, Argentina; 2 Consejo Nacional de Investigaciones Científicas y Técnicas, Instituto de Fisiología, Biología Molecular y Neurociencias (IFIBYNE), Facultad de Ciencias Exactas y Naturales, Universidad de Buenos Aires, CP1428 Buenos Aires, Argentina; 3 Institute of Molecular Biology and Pathology, National Research Council, P.le A. Moro 5, 00185 Rome, Italy; 4 Research Centre for Genomics and Bioinformatics, Council for Agricultural Research and Economics (CREA), Via Ardeatina 546, 00178 Rome, Italy

**Keywords:** Molecular biology, Plant sciences

## Abstract

The germination timing of seeds is of the utmost adaptive importance for plant populations. Light is one of the best characterized factors promoting seed germination in several species. The germination is also finely regulated by changes in hormones levels, mainly those of gibberellin (GA) and abscisic acid (ABA). Here, we performed physiological, pharmacological, and molecular analyses to uncover the role of ATHB2, an HD-ZIP II transcription factor, in germination of *Arabidopsis* seeds. Our study demonstrated that ATHB2 is a negative regulator and sustains the expression of transcription factors to block germination promoted by light. Besides, we found that ATHB2 increases ABA sensitivity. Moreover, ABA and auxin content in *athb2-2* mutant is higher than wild-type in dry seeds, but the differences disappeared during the imbibition in darkness and the first hours of exposition to light, respectively. Some ABA and light transcription factors are up-regulated by ATHB2, such as *ABI5, ABI3, XERICO, SOMNUS* and *PIL5/PIF1*. In opposition, *PIN7*, an auxin transport, is down-regulated. The role of ATHB2 as a repressor of germination induced by light affecting the gemination timing, could have differential effects on the establishment of seedlings altering the competitiveness between crops and weeds in the field.

## Introduction

Seed dormancy is crucial for the adjustment of plant populations to their surrounding environment. Environmental signals that regulate dormancy depth and its alleviation define the germination timing of a seed population and thus are of the outmost adaptive importance^1^. Relevant environmental factors such as temperature, light, and nitrates regulate seed dormancy relief to promote seed germination^2^.

Light has been one of the most characterized factors that regulates seed germination. Phytochromes are well-known photoreceptors that perceive Red (R) and Far-Red (FR) light. They are synthesized in its inactive form, known as Pr (maximum absorption in R), and are photo-converted into its active form, known as Pfr (maximum absorption in FR). *Arabidopsis thaliana* has five phytochromes, known as phyA to phyE. When PHYTOCHROME INTERACTING FACTOR 1 (PIF1), also known as PHYTOCHROME INTERACTING FACTOR 3-LIKE 5 (PIL5), interacts with the Pfr forms of phyA and phyB a massive alteration in the pattern of gene expression in several seed tissues is triggered, influencing the metabolism or sensitivity of several hormones and cell wall proteins, in a direct or indirect manner^3–5^. Gibberellins (GA) promote seed germination and abscisic acid (ABA) represses it. The balance between the content and the hormone sensitivity in the seed tissues, finely regulates the growth of the embryo during the germination process. Environmental signals can regulate this balance by modifying the expression of different metabolic enzymes and the expression of positive and negative regulators of GA and ABA, many of which are feedback regulated^6^.

The *Arabidopsis thaliana* genome encodes around 1500 transcription factors, 40% of which are specific to plants^7^. Homeodomain-Leucine zipper (HD-ZIP) transcription factors are unique to plants and characterized by a HD closely linked to a leucine zipper motif^8^. HD-ZIP proteins constitute a large class of 48 members classified in four distinct families in *Arabidopsis*. Members of the HD-ZIP II protein family are mostly known for their roles in shade avoidance responses^9^, as well as in carpel margin development^10^ and organ polarity^11,12^. In addition, HD-ZIP II protein members control embryonic apical patterning and SAM (shoot apical meristem) function, at least in part, through the interaction with HD-ZIP III proteins^12^. Interestingly, ARABIDOPSIS THALIANA HOMEOBOX2 (ATHB2, also known as HAT4), a member of the HD-ZIP II family, is a direct target of PIF4 and PIF5^13^. In young seedlings and mature plants, the expression of *ATHB2* transcripts is repressed in high R:FR ratios but is rapidly and strongly induced by low R:FR ratios typically found in plant canopy environments through the phytochrome system^14,15^. While seedling hypocotyl growth is activated by ATHB2, primary root growth and lateral root formation are both inhibited by ATHB2, and this is rescued by exogenous auxin^16–18^.

Some previous studies demonstrated that HD-ZIP members can be involved in the regulation of seed germination. For example, *ATHB20*, as a member of the HD-ZIP I family, was also found to be induced by light in seeds, whereas ABA, NaCl or cold treatments had no effect^19^. Moreover, it has been shown that *ATHB20* is strongly expressed in the micropylar endosperm and in the root cap of embryo, acting as positive regulator of seed germination^20^. Another member of this family, *ATHB23*, functions as a positive regulator of the R light-dependent seed germination and mediates cotyledon expansion^21^. Although ATHB2, the most extensively studied HD-ZIP protein, regulates many biological processes controlled by light in plants, its role in seed germination has not been explored. Here, we demonstrated that ATHB2 delays light-induced germination in *Arabidopsis* by sustaining the expression of regulatory genes that increases ABA sensitivity and altering hormonal contents in the seeds.

## Results

### The expression of HD-ZIP II transcription factor genes is altered in light-induced seed germination

We have previously performed RNAseq analyses using imbibed Col-0 seeds in response to a R and FR pulses mediating the promotion and inhibition of germination (85% vs ~ 3%, respectively;^22^). We identified a total of 5785 genes significantly affected by light: 3099 genes were up-regulated whilst 2686 genes were down-regulated (Supplementary Fig. 1). Based on this experiment, we reanalyzed the data for the presence of transcription factor genes that might be involved in the regulation of seed germination; we found that the R light significantly affected the expression of 492 transcription factors genes (~ 8.50% of our transcriptome): 251 genes were up-regulated and 241 genes were down-regulated (Supplementary Fig. 1 and Supplementary Table 1). These genes belong to different families of transcription factors such as HD-ZIP, bHLH, MYB, WRKY and GRAS, among others, and are related to (a) light response: *PIL1*, *PIL2*, *SPT* and *ATHB2*; (b) ABA signaling: *FUS3*, *ABI4* and *ABI5*; (c) GA signaling: *SCL3* and *RGL1*; (d) circadian clock: *PRR1*, *PRR3*, *PRR5*, *PRR7*, *PRR9*, *TOC1*, *LHY* and *CCA1* (Supplementary Table 1).

**Figure 1:**
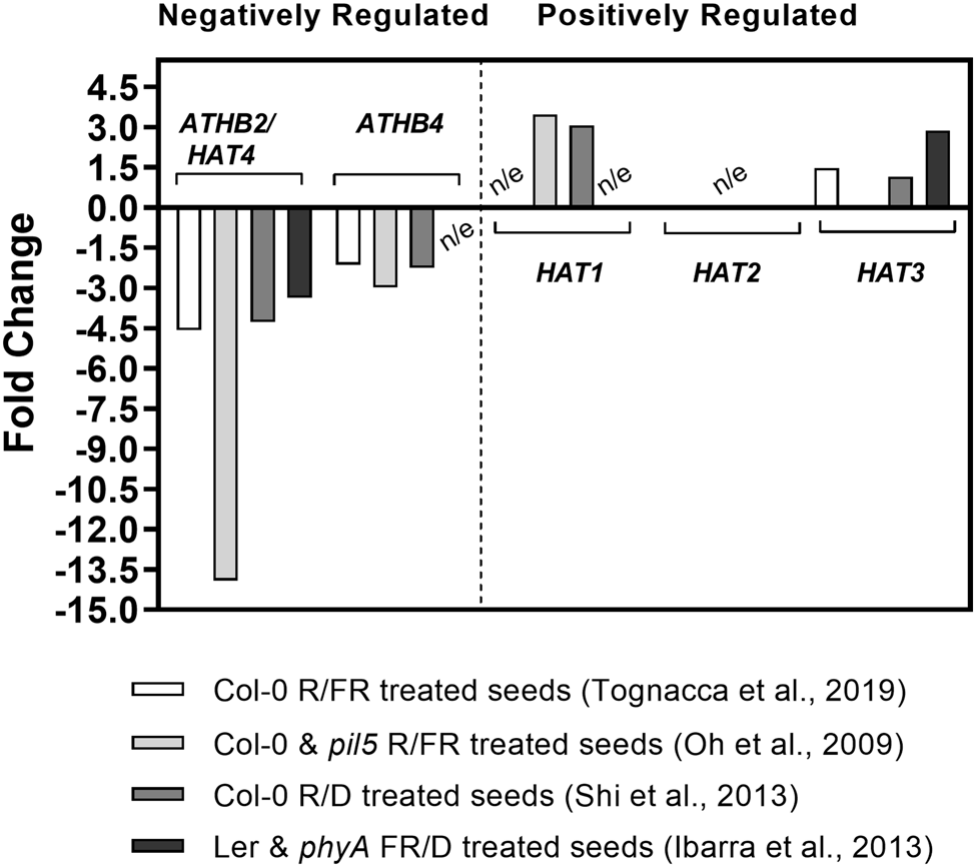
Red light regulates gene expression of HD-ZIP transcription factors in Col-0 seeds. Fold change ratio for HD-ZIP II genes regulated by light in germinating seeds of Arabidopsis in different transcriptomes (phyB-dependent in Shi et al. 2013 and Tognacca et al., 2019; PIL5-dependent in Oh et al. 2009 and phyA-dependent in Ibarra et al., 2013). n/e = no significant expression.

Among the HD-ZIP II transcription factors, *ATHB2* and *ATHB4* were down-regulated whereas *HAT3* was up-regulated by R light, suggesting that HD-ZIP II transcription factors act as negative (ATHB2 and ATHB4) or positive (HAT3) regulators of seed germination induced by light, (Fig. 1 and Supplementary Table 1). Surprisingly, *ATHB2* appeared among the first 20 genes with the highest fold change in our transcriptome (logFC = 4.57). These results are in accordance with previous studies showing that the expression of the *ATHB2* gene and of other members of the HD-ZIP II family is regulated by light during germination through the activity of the phyB^5,22,23^ and the phyA^24^.

**Figure 2:**
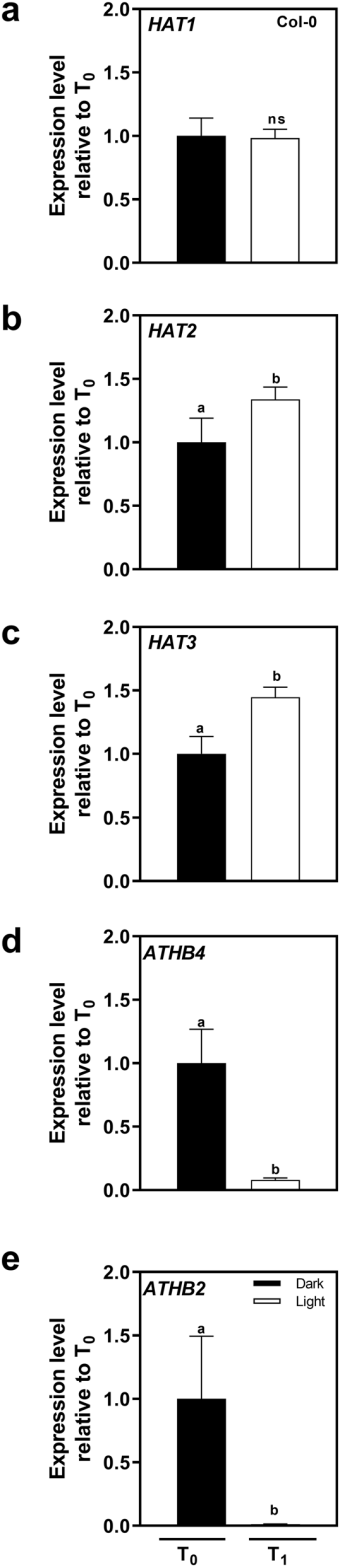
Continuous white light regulates the gene expression of HD-ZIP II members in Col-0 seeds. Seeds were sown for 2 h in darkness (D) and then irradiated with a FRp. Seeds were then incubated for 72 h at 5 °C in D and then incubated at 22 °C under continuous white light (WLc). RNA was extracted from imbibed Col-0 seeds stratified for 3 days at 5 °C and then exposed to WLc for 12 h (defined as T_1_) or kept in darkness prior to the irradiation (defined as T_0_). Gene expression levels of (**a**) *HAT1*, (**b**) *HAT2*, (**c**) *HAT3*, (**d**) *ATHB4* and (**e**) *ATHB2* were normalized to T_0_. Each bar represents mean ± ES (n = 3). Significant differences between means are shown with different letters (*p* < 0.05 by ANOVA followed by Fisher post test).

Since several transcriptomic studies showed that the expression of some members of the HD-ZIP II family can be modulated by white light, we further analyzed the expression of *HAT1*, *HAT2*, *HAT3*, *ATHB2* and *ATHB4* under our experimental conditions by qRT-PCR. RNA was extracted from imbibed Col-0 seeds stratified for 3 days at 5 °C and then exposed to continuous white light (WLc) for 12 h (defined as T_1_). We also harvested a darkness control prior to the irradiation (defined as T_0_). Consistently, white light slightly induced the expression of *HAT2* and *HAT3* and strongly repressed the expression of *ATHB4* and *ATHB2* (Fig. 2), confirming that the expression of different members of HD-ZIP II family can be either induced or repressed during light-mediated seed germination.

### ATHB2 is down-regulated in endosperm cells upon germination under continuous white light

**Figure 3:**
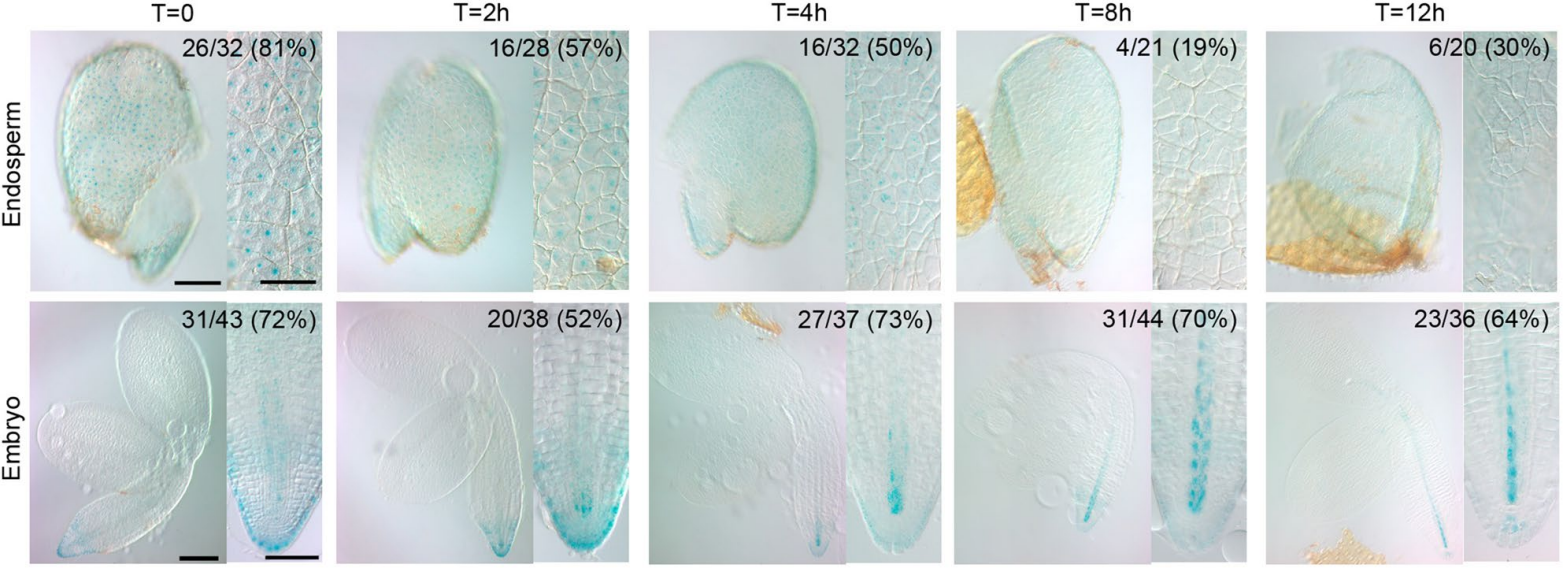
Time-course expression of *ATHB2* under continuous white light. GUS staining of ATHB2:ATHB2:GUS seeds performed at time 0 (T = 0), and after 2 h (T = 2 h), 4 h (T = 4 h), 8 h (T = 8 h), 12 h (T = 12 h) under continuous white light (WLc). Endosperms (Endosperm) and embryos (Embryo) are shown. Insets show an enlarged detail of the same image. Number of the stained samples on the total of the samples are indicated. Percentages are shown in brackets. Bars: Endosperm and Embryo 100 μm; Insets 50 μm.

As *ATHB2* appeared among the first 20 genes with the highest fold change under our experimental conditions, we focused on the role played by ATHB2 in the regulation of seed germination. To this end, we firstly analyzed the localization and distribution of ATHB2:GUS fusion protein in ATHB2:ATHB2:GUS seeds exposed to light. Imbibed seeds were stratified for 3 days at 5 °C and then exposed to continuous white light (WLc) for 12 h. ATHB2:GUS expression was analyzed at four time points between T = 0 (after the stratification and before light irradiation) and T = 12 h (12 h upon light irradiation; Fig. 3). We analyzed separately endosperm sacs and embryos, since discrete roles in the photocontrol of seed germination have been previously shown^25^. At T = 0 h, ATHB2:GUS fusion protein was detected as sharp nuclear staining in endosperm cells (Fig. 3). At the same time, in the embryo ATHB2:GUS expression is also nuclear and is visible in the root tip, mainly in the lateral root cap (LRC), and weakly in the vasculature. At T = 2 h after continuous white light, the signal in the endosperm nuclei is less sharp; in the embryo, the GUS signal is still high in the LRC and expression starts in vascular initial cells. At T = 4 h, the GUS signal in the endosperm is delocalized within the whole cells and the nuclei are hardly recognizable; in the embryo, the signal in LRC decreased while the vascular staining becomes predominant. From T = 8 h to T = 12 h there is a drastic drop of staining in the endosperm cells, as well as in the number of stained endosperm sacs. On the contrary, embryos showed a well-defined change in the ATHB2 pattern, lower in the LRC and higher in the vasculature, with the number of stained embryos remaining constant within 12 h (Fig. 3). We also harvested a darkness control at the end the irradiation, that was similar to the darkness control at T = 0 even at a longer time (Supplementary Fig. 2). Overall, these data suggest that the decrease of ATHB2 expression caused by light during germination is mainly happening in the endosperm.

### ATHB2 delays seed germination under continuous white light

**Figure 4:**
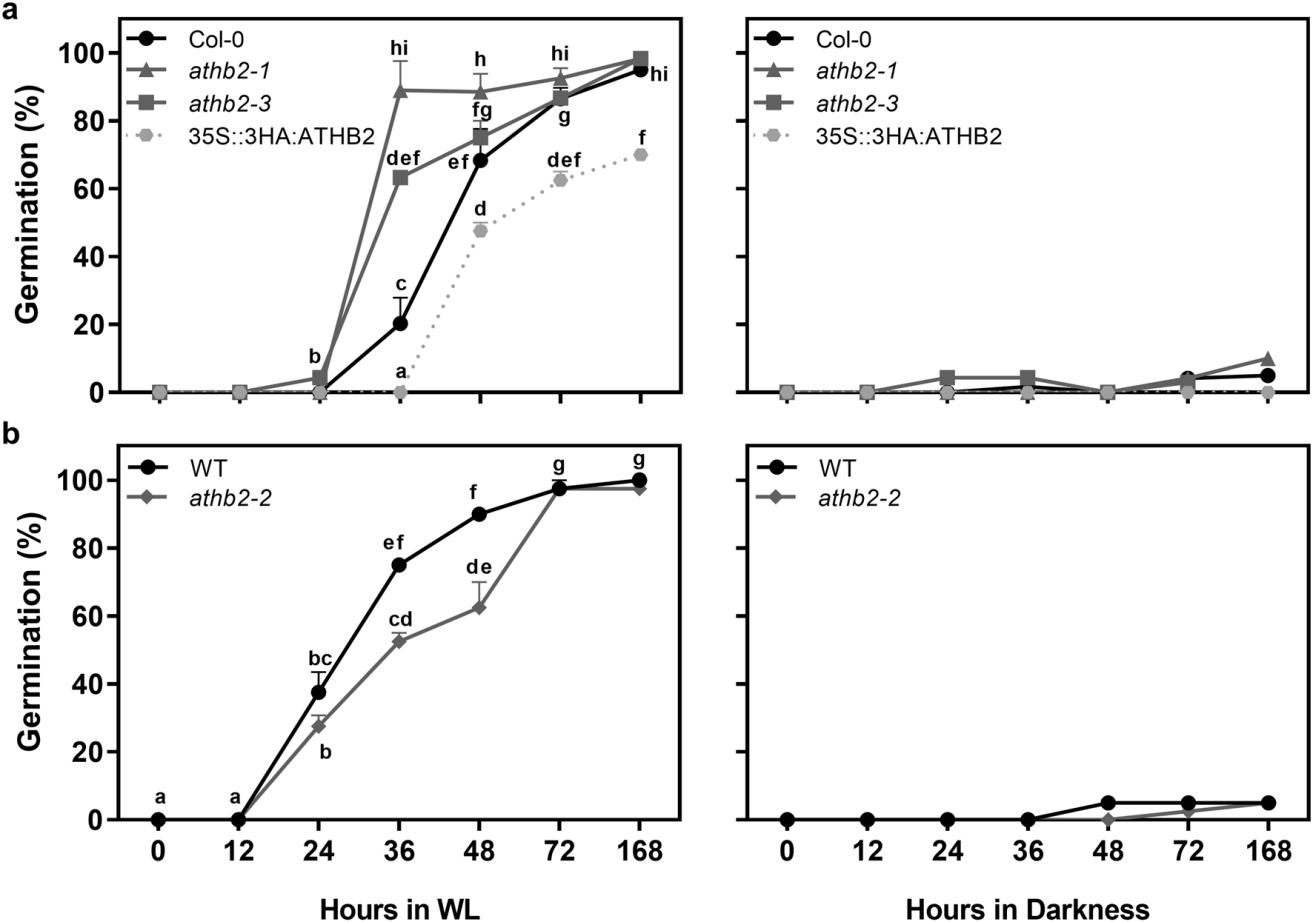
ATHB2 delays seed germination under continuous white light. Seeds were sown for 2 h in darkness (D) and then irradiated with a Far-Red pulse (FRp). Seeds were then incubated for 72 h at 5 °C in D and then incubated at 22 °C under continuous white light (WLc). Germination was counted during variable times upon light irradiation. (**a**,**b**) Germination of Col-0, loss-of-function (*athb2-1*; *athb2-3*) and gain-of-function (35S::3HA:ATHB2; *athb2-2*) seeds incubated for 72 h at 5 °C, previous to light exposure. Right panels represent the darkness control. Each point represents mean ± SE (n ≥ 6). Significant differences between means are shown with different letters (*p* < 0.05 by ANOVA followed by Fisher post test).

We then evaluated the germination of *athb2* mutants under WLc. In particular, we studied the ability to germinate of Col-0, two loss-of-function mutants (*athb2-1* and *athb2-3*) and two of gain-of-function lines (35S::3HA:ATHB2 and *athb2-2*). To this end, seeds were incubated in darkness at 5 °C for three days prior to WLc irradiation at 22 °C. Seed germination time-course was evaluated upon light treatment for 7 days (Fig. 4). Col-0 seeds started to germinate around 24 h reaching maximum germination at the 7th day (i.e., at 168 h ~ 90%; Fig. 4a). Loss-of-function *athb2* seeds fully germinated (*athb2-1*) or accelerated the process germinating ~ 70% (*athb2-3*) at 36 h. On the contrary, seeds of the *athb2-2* gain-of-function mutant delayed the germination (Fig. 4b) and the over-expressing 35S::3HA:ATHB2 line also reduced the final germination compared to wild-type (Fig. 4a). Dark germination at the end of the experiment was ~ 0% for all genotypes (Fig. 4, right pannels).

### ATHB2 controls the concentration of ABA and auxin during seed germination

**Figure 5:**
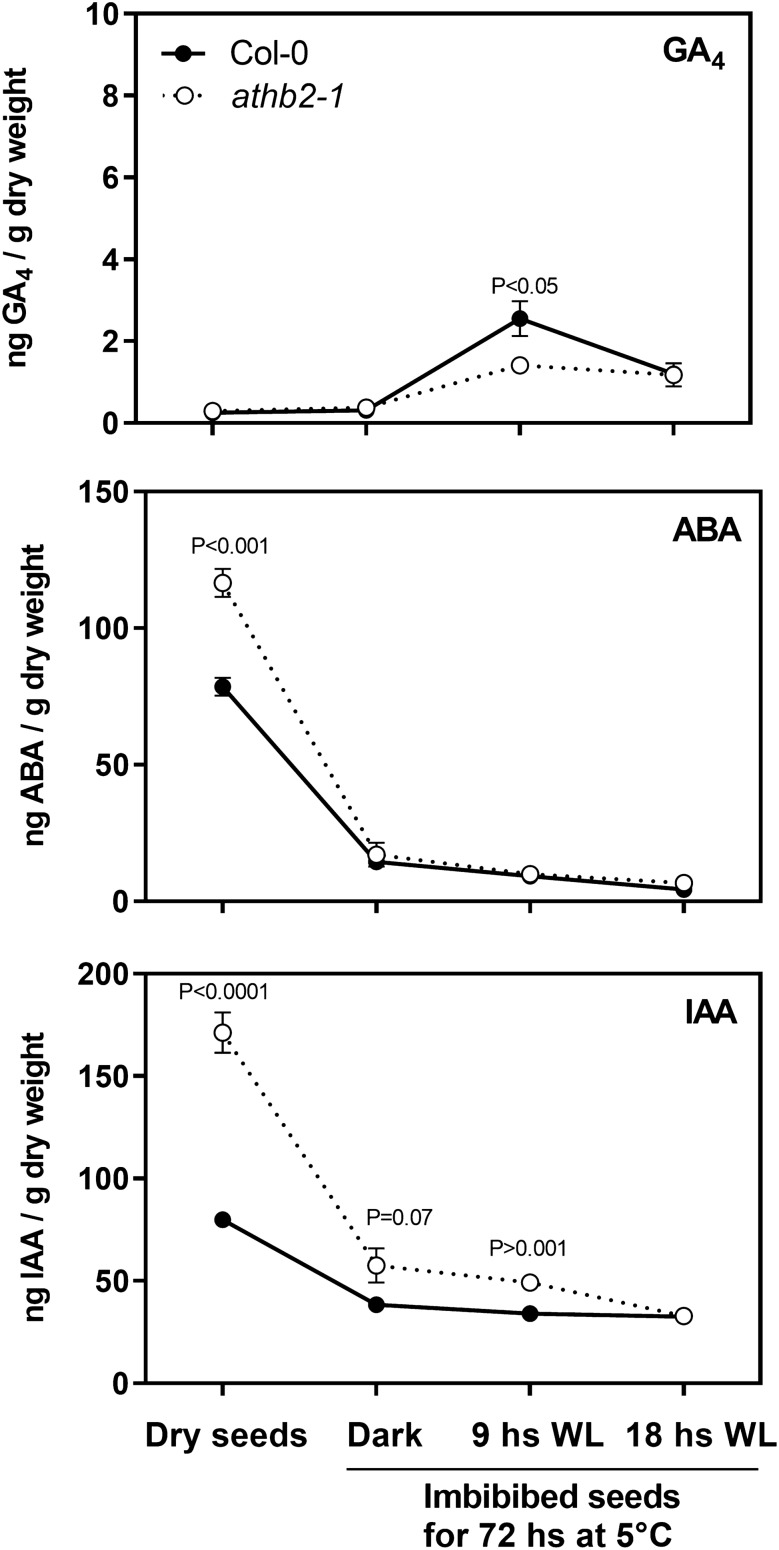
ATHB2 reduces IAA content of the seeds. GA_4_, ABA and IAA levels in Col-0 and *athb2-1* seeds incubated in darkness at 5 °C for 3 days days prior to irradiation under continuous white light (WLc) at 22 °C. (**A**) mature dry seeds. (**B**) 72 h stratified imbibed seeds in darkness (prior to light irradiation). (**C**) 72 h stratified imbibed seeds in darkness and then irradiated for 9 h under WLc. (**D**) 72 h stratified imbibed seeds in darkness and then irradiated for 18 h under WLc. Significant differences between means are shown with different letters (*p* < 0.05 by ANOVA followed by Fisher post test).

We quantified GA_1_, GA_4_, ABA and IAA levels in Col-0 and *athb2-1* seeds incubated in darkness at 5 °C for 3 days days prior to irradiation with WLc at 22 °C. We measured hormone levels at four time points: (a) mature dry seeds, (b) 72 h stratified imbibed seeds in darkness (prior to light irradiation), (c) 72 h stratified imbibed seeds in darkness and then irradiated for 9 h with WLc and (d) 72 h stratified imbibed seeds in darkness and then irradiated for 18 h with WLc. GA_4_ is slightly increased in stratified seeds irradiated for 9 h with white light, both in Col-0 and *athb2-1* seeds (Fig. 5) whilst GA_1_ levels were not affected neither by the time point nor by the genotype (Supplementary Fig. 3), supporting the idea that GA synthesis is not clearly related to the ATHB2 function during seed germination.

ABA levels were higher in *athb2-1* compared to Col-0 in dry seeds (Fig. 5), and then similar ABA levels were found between both genotypes in stratified seeds in darkness (Fig. 5). IAA content was higher in *athb2-1* compared to Col-0 in dry seeds (Fig. 5) as well as in imbibed seeds stratified for 72 h in darkness (Fig. 5) or irradiated for 9 h with WLc after stratification (Fig. 5). These results suggest that ATHB2 can affect the accumulation of ABA and auxin levels in the dry seeds and during the imbibition in darkness at low temperatures as well as after the first hours of light irradiation in the case of IAA.

### ATHB2 positively affects ABA signaling during seed germination

**Figure 6:**
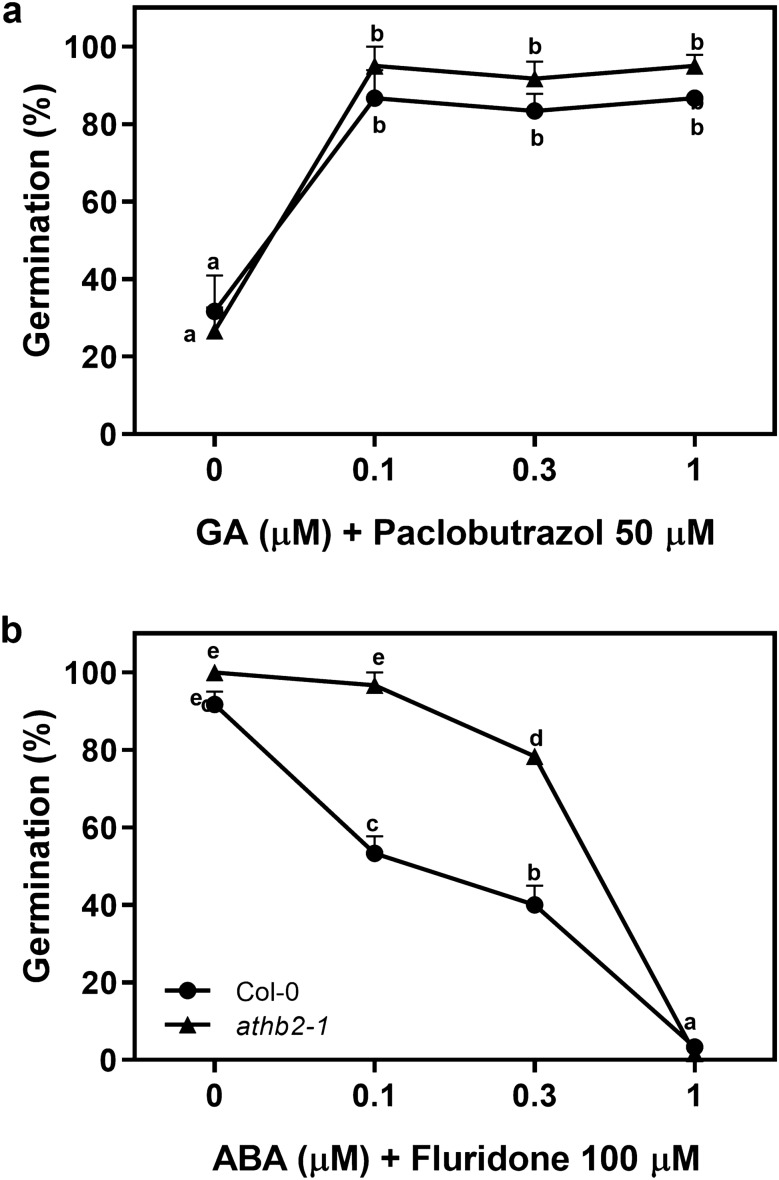
ATHB2 increases the ABA sensitivity of the seeds. Germination of Col-0 and *athb2-1* seeds incubated for 72 h at 5 °C, previous to light exposure. Seeds were imbibed with increasing doses of (**a**) GA_4_, in the presence of 50 uM paclobutrazol; and (**b**) ABA, in the presence of 100 uM fluridone. Each point represents mean ± SE (n ≥ 6). Significant differences between means are shown with different letters (*p* < 0.05 by ANOVA followed by Fisher post test).

As GA and ABA are the most relevant hormones regulating seed germination, we analyzed dose-dependent germination curves responses of Col-0 and *athb2-1* seeds incubated with inhibitors and/or synthetic hormones. We evaluated the sensitivity of the seeds to GA by adding increasing doses of GA_4_ in a medium supplemented with 50 µM paclobutrazol, which inhibits endogenous GA biosynthesis. Col-0 and *athb2-1* seeds germinated at the same rate in all the doses evaluated, ranging from 0 µM to 1 µM. The germination was promoted between ~ 20% and ~ 95% (Fig. 6a). We also evaluated the sensitivity of the seeds to ABA in a medium containing increasing doses of ABA supplemented with 100 µM fluridone, which inhibits endogenous ABA biosynthesis. Whereas the addition of fluridone in a medium without ABA did not affect the germination of Col-0 and *athb2-1* seeds (~ 92% and 100%, respectively; Fig. 6b), increasing the concentration of ABA in the medium reduced the germination of the seeds. In fact, the addition of 0.1 µM ABA inhibits by ~ 40% the germination of Col-0 seeds, while it did not significantly affect the germination of *athb2-1* seeds. Interestingly, the germination of Col-0 seeds was significantly lower than *athb2-1* seeds (40% vs. 80%, respectively) at ABA concentration equal to 0.3 µM. Seeds of both genotypes did not germinate with 1 µM ABA (Fig. 6b). We conclude that ATHB2 affects ABA sensitivity without altering GA signaling in imbibed seeds during germination.

Finally, we evaluated the germination of *athb2-1* and Col-0 seeds imbibed in a medium with the addition of Picloram (PIC), a synthetic auxin, or 1-NOA, an auxin influx and efflux inhibitor. In both cases, increasing doses of PIC or 1-NOA reduced the germination of both genotypes at similar levels. Doses of 1 mM PIC reduced the germination ~ 20% and 10 mM PIC led to complete inhibition of germination in both genotypes (Supplementary Fig. 5). Adding 1 µM 1-NOA did not change the germination of Col-0 nor *athb2-1* seeds; while adding 200 µM 1-NOA reduced the germination of Col-0 and *athb2-1* in similar proportion. Higher doses of 1-NOA equal to 500 µM led to complete inhibition of germination in both genotypes (Supplementary Fig. 5).

### ATHB2 promotes gene expression of negative regulators of seed germination

**Figure 7:**
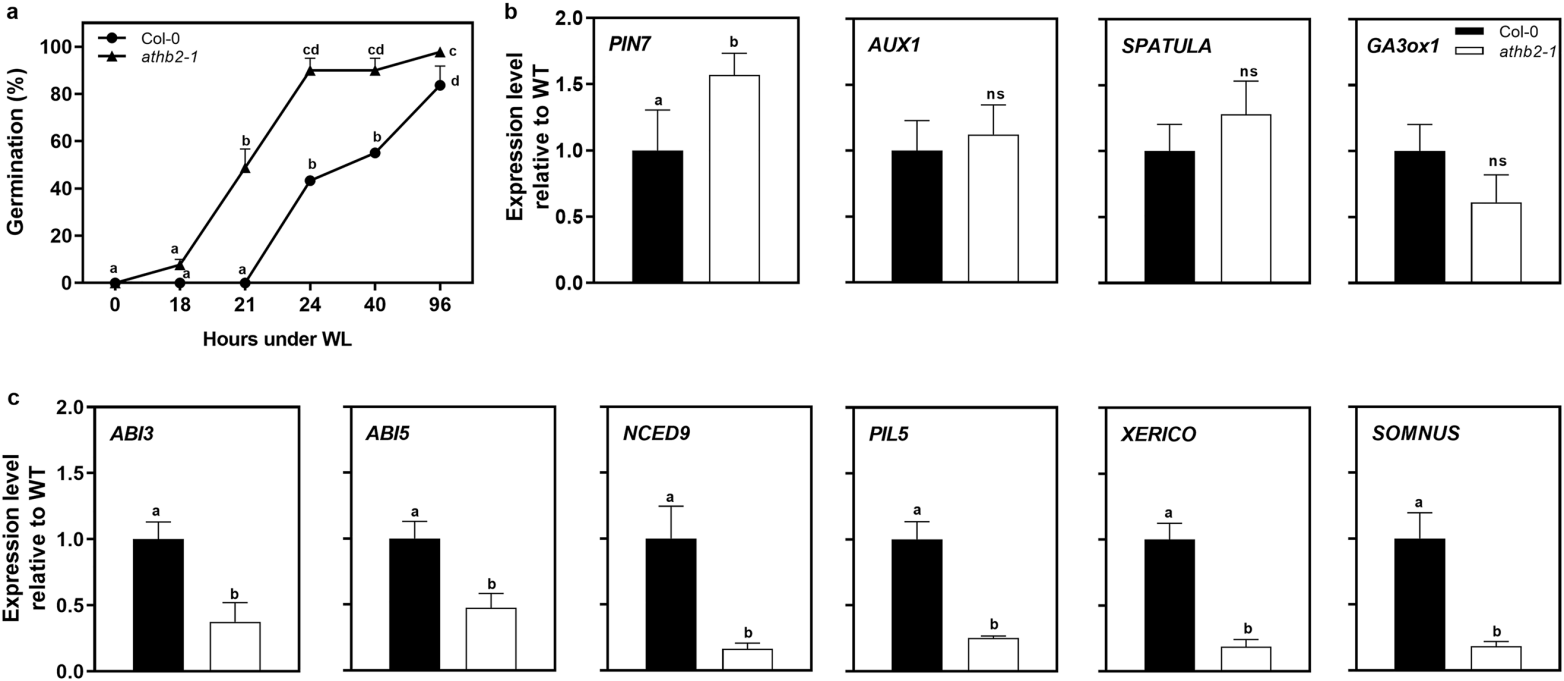
ATHB2 regulates the expression of negative regulators, ABA signaling and auxin transporter genes in germinating seeds. (**a**) Seeds were sown for 2 h in darkness and then irradiated with a Far-Red pulse (FRp). Seeds were then incubated for 72 h at 5 °C in darkness (D) and then incubated at 22 °C under continuous white light (WLc). Germination was counted during variable times upon light irradiation. Germination of Col-0 and *athb2-1* incubated for 72 h at 5 °C, previous to light exposure, is shown. Each point represents mean ± SE (n ≥ 3). (**b**) Genes repressed or non-regulated by ATHB2. (**c**) Genes induced by ATHB2. In (**B**) and (**C**), Col-0 and *athb2-1* seed samples were harvested after 12 h under WL. Gene expression levels were normalized to Col-0. Each bar represents mean ± ES (n = 3). Significant differences between means are shown with different letters (*p* < 0.05 by ANOVA followed by Fisher post test).

In order to have a better understanding of the molecular mechanisms underlying ATHB2-related seed germination, we analyzed the expression of genes belonging to different signaling pathways (auxin, GA and ABA). We collected Col-0 and *athb2-1* samples for RNA isolation after 12 h in WLc (Fig. 7a). Since some members of the HD-ZIP II family can regulate plant development through the auxin signaling^12,16^, we evaluated the expression of some auxin-related components in the seeds. *AUX1* expression, an influx auxin carrier, did not change in *athb2-1* when compared to Col-0 seeds, while the expression of *PIN7*, an efflux auxin carrier, was significantly induced (Fig. 7b). These results suggest that ATHB2 down-regulates *PIN7* expression. The expression of *SPATULA*, a light-stable repressor of the thermo-response in seeds, and *GA3ox1*, an enzyme involved in the synthesis of GA, did not change in *athb2-1* compared to Col-0 seeds (Fig. 7b).

Finally, we observed a generalized pattern of repression of ABA and light-related genes in *athb2-1* compared to Col-0 seeds (Fig. 7c). *ABI3* and *ABI5*, regulators of ABA signaling, and *NCED9*, a key enzyme in the biosynthesis of ABA, were significantly less expressed in *athb2-1* seeds than in Col-0 seeds (Fig. 7c). *PIF1/PIL5*, *XERICO* and *SOMNUS* expression were also significantly decreased in *athb2-1* compared to Col-0 seeds (Fig. 7c). We conclude that an accelerated germination of *athb2-1* seeds correlates well with a decrease in the gene expression of transcription factor related with ABA and light signaling that act as repressors of germination in *Arabidopsis*.

## Discussion

Seed germination is a process of extreme relevance for a successful seedling establishment in the field. The information concerning transcriptional and gene expression regulation of light-induced seed germination is abundant^4,5,22,26–29^. By comparative transcriptomic analysis, we identified ATHB2 as a negative regulator of light-induced seed germination. We showed that a pulse of R light can affect the expression of ~ 20% of *Arabidopsis* transcriptome in Col-0 imbibed seeds (5785 genes, Supplementary Fig. 1 and Supplementary Table 1) and ~ 8.50% of those 492 genes are transcription factors (Supplementary Fig. 1,^22^). Interestingly, *ATHB2* appeared among the first 20 down-regulated genes with the highest fold change^22^. Previous studies suggested that both phyA and phyB regulate the expression of *ATHB2* in imbibed *Arabidopsis* seeds (Fig. 1;^5,22,24^). Interestingly, different members of the HD-ZIP II class show opposite expression patterns. Some HD-ZIP II transcription factors are promoted (*HAT1*, *HAT2* and *HAT3*), whilst others are repressed (*ATHB2* and *ATHB4*) in *Arabidopsis* seeds (Fig. 1). In particular, white light represses *ATHB2* expression within 12 h after induction of seed germination (Fig. 2). In these conditions, we also showed that the ATHB2 expression decreases specifically in the endosperm and in the embryonic root cap cells, whereas it increases in the vasculature of the embryo (Fig. 3). Since the ABA produced in the endosperm is critical for seed dormancy establishment and germination, we can also not discard differentially expression of ATHB2-regulated gene expression in embryo and endosperm tissues. The endosperm has been previously shown to be responsible for the inhibition of embryo germination in *Arabidopsis* imbibed dormant seeds, actively producing and releasing ABA^30^. Furthermore, phytochromes control of seed germination also acts in separate times and compartments, with FR light initially repressing germination of the embryo through phyB contained in the endosperm, and later sustaining a phyA-dependent germination from the embryo. This involves ABA release from the endosperm and distinct spatial activities of phytochrome signaling components^25^. The observed higher expression of ATHB2 in the endosperm of stratified seeds, just before white light-induced seed germination, and its rapid downregulation within the first hours of light exposure, is therefore suggestive that the negative role exerted by the endosperm on seed germination requires ATHB2 (Fig. 3). However, ATHB2 is also expressed in the vasculature during the germination, where it appears to be positively regulated by light (Fig. 3), suggesting that ATHB2 could have a different role in this tissue, likely more linked to auxin signaling preparing for post-germination events associated with seedling de-etiolation (see below). In the future, it will be interesting to study in detail the specific roles of ATHB2 in the endosperm and in the embryo, respectively.

ABA accumulation in dry seeds has been observed in both dormant and non-dormant *Arabidopsis* seeds, and it rapidly decreases upon imbibition in both cases^31,32^. The difference between dormant and non-dormant seeds has been shown to lie primarily in their ability to synthesize de novo ABA after imbibition^31^. However, the higher concentration of ABA observed in *athb2-1* mutant dry seeds has no effect on germination after imbibition (Fig. 5). Conversely, ABA levels in *athb2-1* mutant decrease to amounts comparable to those in the wild type after imbibition (Fig. 5). Therefore, the reduced levels of ABA signaling regulators observed after 12 h of imbibition under WLc suggest a role of ATHB2 in this process downstream or independent of ABA. In this plausible scenario, ATHB2 might be responsible -directly or indirectly- for the higher levels of ABA observed in *athb2-1* dry seeds (Supplementary Fig. 6). Accordingly, Stamm et al. (2012) have shown that *ATHB2* appears to be highly expressed in seed tissues^33^. Moreover, analyses of different transcriptomes during seed development in *Arabidopsis* also show that *ATHB2* is expressed in embryos^34^, dry seeds^35^ and in seeds across different time-courses during germination^36^. It has been reported that HAT1 and its homolog HAT3 redundantly and negatively regulate both ABA biosynthesis and signaling genes in a complex transcriptional regulation^37^. A similar regulatory circuit might be operating during seed maturation through ATHB2. Indeed, we have shown that ATHB2 exerts its function in several processes in combination with at least HAT3 and ATHB4^12,38^. Further work needs to be done to understand the role of ATHB2 during seed development and/or after-ripening.

Recently, it has been shown that *pif1/pif5* and the quadruple *pif1*/*pif3*/*pif4*/*pif5* are insensitive to ABA whereas *phyB* mutant displayed hypersensitivity to ABA in seed germination, respectively, reinforcing the hypothesis that the PIFs inhibit germination by modulating ABA signaling^39^. Interestingly, here we show that the lack of ATHB2 results in a lower expression of *PIF1/PIL5* and *SOM* genes respect to the control (Fig. 7). As PIF1/PIL5 positively regulates *ATHB2* expression^5,23,24^, our results suggest that ATHB2 might be involved in a positive feedback loop with PIF1/PIL5, which in turn control the expression of SOM^40^, thus enhancing the repressor activities in seed germination mediated by ABA. Accordingly, the lack of ATHB2 result in a lower expression of genes encoding relevant components of the ABA signaling such as those of *ABI3*, *ABI5*, *NCED9*, *XERICO* and *SOM* (Fig. 7). XERICO promotes *ABI3* expression^41^, a transcription factor that is involved in the ABA signaling; and SOM promotes the expression of *NCED9*^42^, a gene involved in the ABA synthesis. In agreement with that, the ABA sensitivity (Fig. 6) and the ABA signaling components (Fig. 7) of the seeds are also reduced in *athb2-1* mutant. Therefore, our results suggest that ATHB2 acts as a negative regulator of the light-dependent germination, exerting its function by affecting ABA signaling. Therefore, we suggest that ATHB2 acts as a negative regulator of the light-dependent germination, exerting its function by affecting ABA signaling. Interestingly, similar roles for other HD-ZIP members during seed germination have also been established. Barrero et al. (2010) have shown that a member of the HD-ZIP I family, ATHB20, whose expression is also regulated by light, promotes germination in *Arabidopsis* seeds. *ATHB20* is expressed in the micropylar endosperm and in the root cap, and reduces ABA sensitivity^20^. ATHB23, another member of the same family as ATHB20, has also been associated with the promotion of seed germination^21^. Based on our results and those on ATHB20, we can speculate that ATHB2 and ATHB20 play different and antithetical roles at least in the root cap where they are oppositely regulated by light.

Links between HD-ZIP II proteins and auxin have been established^43,44^. However, the molecular mechanisms involved between HD-ZIP II transcription factors and the auxin signaling is still poorly understood. Our analysis found that ATHB2 controls the IAA production in dry seeds and during a short period of seed imbibition (up to 9 h upon light irradiation, Fig. 5). We also demonstrated that ATHB2 negatively affects at least the expression of the auxin transporter *PIN7* in light-induced germination (Fig. 7). The transcriptome induced by phytochromes in seeds showed that auxin transport proteins are a pre-requisite for germination^5,22–24^. PIN7 has been involved in the pattern specification during root development, being expressed at lateral and basal membranes of provascular cells in the meristem and elongation zone^45^. Then, the higher expression of *PIN7* in the *athb2-1* mutant might produce positive effects on proliferation and cellular elongation of the embryonic axis during germination, thus accelerating the emergence of the embryonic radicle.

In conclusion, our work suggests that ATHB2 is a negative regulator of light-mediated germination that control a myriad of ABA and light-related transcription factors by reducing ABA sensitivity. The knowledge generated in this work using *Arabidopsis* seeds could be potentially expanded to other species in the future, especially crop plants, to decipher the molecular networks for the relief of dormancy by light. Furthermore, the identification of ATHB2 as a repressor that delays the timing of seed germination could have effects on the establishment of seedlings changing the competitiveness between crops and weeds in the field.

## Materials and methods

### Plant material and growth conditions

*Arabidopsis thaliana* plants were grown as previously described in^22^. Plants were grown under long day conditions (16 h light/8 h dark, PAR = 100 µmol m^−2^ s^−1^) with an average temperature of 21 ± 2 °C. Wild-type and mutant plants were grown together and their mature seeds were harvested at the same time to avoid differences in post-maturation that can affect seed germination. Seeds of each genotype were harvested as a single bulk consisted of at least 5 plants. Seeds were stored in open tubes inside a closed box and maintained in darkness with silica gel at 4 °C until the experiments were performed. *Arabidopsis thaliana* wild-type Columbia-0 (Col-0) seeds were obtained from the Arabidopsis Biological Resource Centre. In this study, we used insertional knock-out lines: *athb2-1* (Salk_106790;^46^), and *athb2-3*^12^; an over-expressing line: *athb2-2* (Salk_006502,^12^) and two over-expressing tagged lines: ATHB2::ATHB2:GUS^12^ and 35S::3HA:ATHB2^12^.

### Germination conditions and light treatments

Samples of 30 seeds per genotype were sown in clear plastic boxes, each containing 10 mL of 0.8% (w/v) agar in demineralized water. To establish a minimum and equal photo-equilibrium, seeds were imbibed for 2 h in darkness and then irradiated for 20 min with a saturated far-red (FR) pulse (calculated Pfr/P = 0.03, 42 µmol m^−2^ s^−1^) in order to minimize the quantities of Pfr formed during their development in the mother plant. Seeds were then stratified at 5 °C in darkness for 3 days and transferred to continuous white light at 22 °C. Germination was counted during variable time upon light irradiation until the day 7. The criterion for germination was the emergence of the radicle.

For the hormone experiments, seeds were sown in clear plastic boxes, each containing filter papers imbibed with 750 µL of ABA or GA (0.1, 0.3 and 1 µM) supplemented with fluridone 100 µM (Sigma‐Aldrich, Steinheim, Germany) or paclobutrazol 50 µM (Sigma‐Aldrich, Steinheim, Germany) until the end of the experiment, respectively. We have previously performed calibration curves to determine the minimum concentration of paclobutrazol required to completely inhibit or fluridone required for maximum promotion of seed germination (Supplementary Fig. 4). To test the auxin sensitivity of the seeds, seeds were sown in clear plastic boxes, each containing 750 µL of picloram (1, 10 and 100 mM) or 1-NOA (1, 200 and 500 µM) (Sigma‐Aldrich, Steinheim, Germany).

### Transcriptomic analyses

For Fig. 1, Fold Change values are the same as in the corresponding original works. The RNA-seq data from Tognacca et al., 2019 is available at the Gene Expression Omnibus (GEO) with this accession: GSE134019. Differentially expressed genes were identified using the edgeR v3.4.2 package with FDR < 0.05 (false discovery rate). Genes with a positive logFC value were classified as down-regulated and genes with a negative logFC value as up-regulated under red light (Tognacca et al. 2019). For Oh et al. 2009 we used the “List of PIL5 regulated genes” dataset. For Shi et al. 2013 we used the “WT-R vs WT-D diffgenes” dataset. For Ibarra et al. 2013 we used the “phyA dependent genes” dataset regarding the late-down and late-up classification (5 h after FR pulse).

### Gene expression analysis by quantitative RT–PCR

We followed our protocol described in^22^. Seed samples (~ 10 mg seed dry weight) were sown in clear plastic boxes, each containing 10 mL of 0.8% (w/v) agar in de-mineralized water and incubated for three days in darkness at 5 °C. Seeds were then exposed to white light at 22 °C and samples were collected 12 h upon light irradiation. After sampling, seeds were immediately frozen in liquid nitrogen and stored at  − 80 °C. RNA was extracted using the Spectrum Plant Total RNA Kit (Sigma-Aldrich, Steinheim, Germany) according to manufactures protocol. cDNA was synthesized using MMLV High Performance Reverse Transcriptase (Epicentre, Madison, USA) and oligo-dT primers. The synthesized cDNAs were amplified with FastStart Universal SYBR Green Master (Roche, Madison, USA) using the 7500 Real Time PCR System cycler (Applied Biosystems, Foster City, CA, USA). PP2A gene was used as a normalization control. The primers used are described in Supplementary Table 2.

### Quantification of hormone levels

The thoroughly ground tissue (~ 30–50 mg seed dry weight) was suspended in 80% methanol  − 1% acetic acid containing internal standards and mixed by shaking during one hour at 4 °C. The extract was kept a  − 20 °C overnight and then centrifuged and the supernatant dried in a vacuum evaporator. The dry residue was dissolved in 1% acetic acid and passed through a reverse phase column (HLB Oasis 30 mg, Waters), as described in^47^. The final residues were dried and dissolved in 5% acetonitrile  − 1% acetic acid and the hormones were separated by UHPLC with a reverse Accucore C18 column (2.6 µm, 100 mm length; Thermo Fisher Scientific) with a 2 to 55% acetonitrile gradient containing 0.05% acetic acid, at 400 µL/min over 21 min. The hormones were analyzed with a Q-Exactive mass spectrometer (Orbitrap detector; Thermo Fisher Scientific) by targeted Selected Ion Monitoring (tSIM; capillary temperature 300 °C, S-lens RF level 70, resolution 70.000) and electrospray ionization (spray voltage 3.0 kV, heater temperature 150 °C, sheath gas flow rate 40 µL/min, auxiliary gas flow rate 10 µL/min) in negative mode. The concentrations of hormones in the extracts were determined using embedded calibration curves and the Xcalibur 4.0 and TraceFinder 4.1 SP1 programs. The internal standards for quantification of each of the different plant hormones were the deuterium-labelled hormones (purchased from OlChemim Ltd, Olomouc, Czech Republic). The hormone quantification analysis was carried out at the Plant Hormone Quantification Service, IBMCP, Valencia, Spain.

### GUS analysis

GUS analyses were performed as described^48^. In brief, seeds were fixed in cold 90% acetone for 1 h. During fixation, the testa was peeled off the seed and the endosperm was separated from embryo under a stereomicroscope. Endosperms and embryos were washed twice in 100 mM phosphate buffer. The material from the same time point was then stained in the same staining solution at 37 °C for 24 h. Staining was stopped in 80% EtOH, and then samples were mounted in chloral hydrate for DIC analysis under an Axioscop 2 Plus (Zeiss, Germany) binocular microscope. Experiments were performed four times with two independent lines.

### Experimental design and statistical analysis

Physiological and hormonal experiments were done using at least three different populations of seeds harvested for at least 6 plants. To test for significant differences in the response of the seeds, we conducted two-way ANOVAs for each wild-type and mutant group, using the angular transformation of the percentage of germination and the InfoStat Software version 2017 (Grupo InfoStat, FCA, Universidad Nacional de Córdoba, Argentina). Fisher post-test was used to test differences when significant interactions were observed.

All the experiments and collection are done in accordance with the relevant institutional, national, and international guidelines and legislation. We have also obtained the corresponding permission to collect *Arabidopsis thaliana* seeds and plant material.

## Supplementary Information

Supplementary Information 1.

Supplementary Information 2.

Supplementary Information 3.

Supplementary Information 4.

Supplementary Information 5.

Supplementary Information 6.

Supplementary Information 7.

Supplementary Information 8.

Supplementary Information 9.
