# Author Correction: ATHB2 is a negative regulator of germination in *Arabidopsis thaliana* seeds

**DOI:** 10.1038/s41598-021-91955-0

**Published:** 2021-06-14

**Authors:** Rocío Soledad Tognacca, Monica Carabelli, Giorgio Morelli, Ida Ruberti, Javier Francisco Botto

**Affiliations:** 1 Consejo Nacional de Investigaciones Científcas y Técnicas, Instituto de Investigaciones Fisiológicas y Ecológicas Vinculadas a la Agricultura (IFEVA), Facultad de Agronomía, Universidad de Buenos Aires, Av. San Martín 4453, C1417DSE Buenos Aires, Argentina; 2 Consejo Nacional de Investigaciones Científcas y Técnicas, Instituto de Fisiología, Biología Molecular y Neurociencias (IFIBYNE), Facultad de Ciencias Exactas y Naturales, Universidad de Buenos Aires, CP1428 Buenos Aires, Argentina; 3 Institute of Molecular Biology and Pathology, National Research Council, P.le A. Moro 5, 00185 Rome, Italy; 4 Research Centre for Genomics and Bioinformatics, Council for Agricultural Research and Economics (CREA), Via Ardeatina 546, 00178 Rome, Italy

Correction to: *Scientific Reports* 10.1038/s41598-021-88874-5, published online 06 May 2021

The original version of this Article contained errors in the spelling of the authors Rocío Soledad Tognacca, Monica Carabelli, Giorgio Morelli, Ida Ruberti & Javier Francisco Botto which were incorrectly given as Tognacca Rocío Soledad, Carabelli Monica, Morelli Giorgio, Ruberti Ida & Botto Javier Francisco.

The original Article has been corrected.

