# Supplementary material for: ATHB2 is a negative regulator of germination in Arabidopsis thaliana seeds
Source: Sci Rep. 2021 May 6;11:9688. doi: 10.1038/s41598-021-88874-5 (PMC8102570; doi:10.1038/s41598-021-88874-5)
Supplement: Supplementary file 1 — Supplementary Information 1. [file 41598_2021_88874_MOESM1_ESM.docx]

**Supporting Information**

**Supplementary Figure 1.** **(a)** Experimental protocol used to assay seed germination and transcriptome profile in Col-0 seeds. Seeds were sown for 2 h in darkness (D) and then irradiated with a far-red pulse (FRp). Seeds were then incubated for 72 h at 5 °C in D and finally irradiated with a red pulse (Rp) or FRp. Germination was counted after 72 h at 25 °C. **(b)** A total of 5785 genes were differentially expressed (DE) under R light. ~ 8.50 % (492 genes) belongs to different transcription factors families. Data from Figures A-B are from Tognacca *et al*., 2019.

**Supplementary Figure 2.** Time course expression of ATHB2 under 24 hours continuous white light. GUS staining of ATHB2:ATHB2:GUS seeds performed at time 0 (T = 0), and after 2 hours (T = 2 h), 4 hours (T = 4 h), 8 hours (T = 8 h), 12 hours (T = 12 h), 24 hours (T = 24 h) under continuous white light (WLc). A control harvested after 24 hours in darkness was also collected (T = 24D). Endosperms (Endosperm) and embryos (Embryo) are shown. Insets show an enlarged detail of the same image. Number of the stained samples on the total of the samples are indicated. Percentages are shown in brackets. Bars: Endosperm and Embryo 100 μm; Insets 50 μm. Panels **(a)** and **(b)** correspond to two different lines.

**Supplementary Figure 3.** GA_1_ hormone quantification in seeds of Col-0 and *athb2-1.*

**Supplementary Figure 4.** Calibration curves for the hormonal experiments.

**Supplementary Figure 5.** ATHB2 is not involved in the auxin sensitivity of the seeds.

**Supplementary Figure 6.** Expression of *ATHB2* transcripts during **(a)** seed development and **(b)** seed germination. Data for seed development was obtained from Gene Expression Map of Arabidopsis Development (Nakabayashi et al., 2005; Schmid et al., 2005 [49]). Gene expression data generated by the Affymetrix ATH1 array are normalized by the GCOS method, TGT value of 100. The RNAseq data for germination was obtained from Narsai et al., 2017. The original images were generated with the AtGenExpress eFP at bar.utoronto.ca/eplant by Waese et al., 2017 [50]. Expression data were taken from <http://bar.utoronto.ca/eplant/>.

**Supplementary Table 1**. List of transcription factors significantly affected by light.

**Supplementary Table 2.** Primers used in the study*.*
