# Supplementary figures and images for: ATHB2 is a negative regulator of germination in Arabidopsis thaliana seeds
Source: Sci Rep. 2021 May 6;11:9688. doi: 10.1038/s41598-021-88874-5 (PMC8102570; doi:10.1038/s41598-021-88874-5)

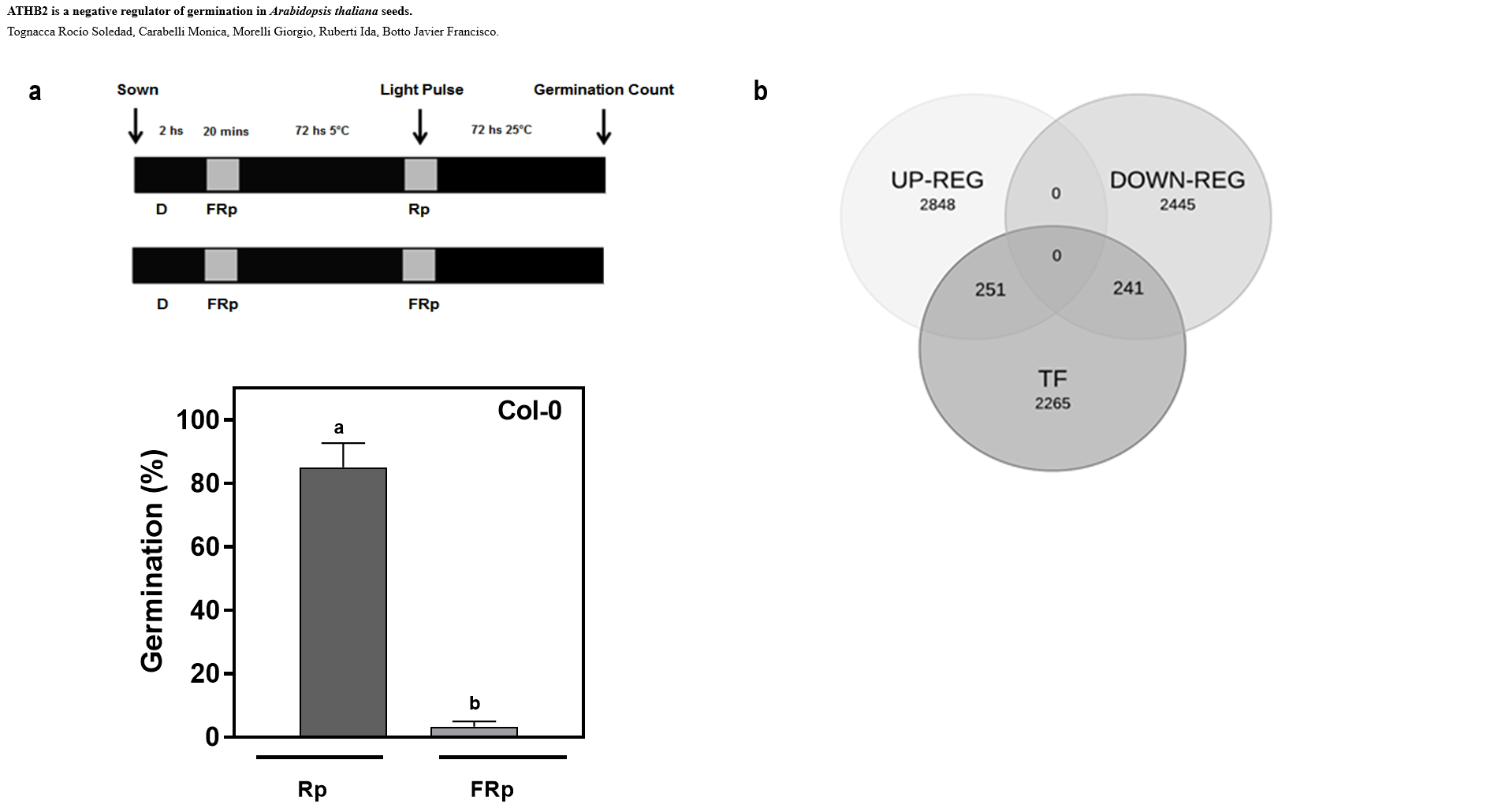

Supplement: Supplementary file 2 — Supplementary Information 2. [file 41598_2021_88874_MOESM2_ESM.tiff]

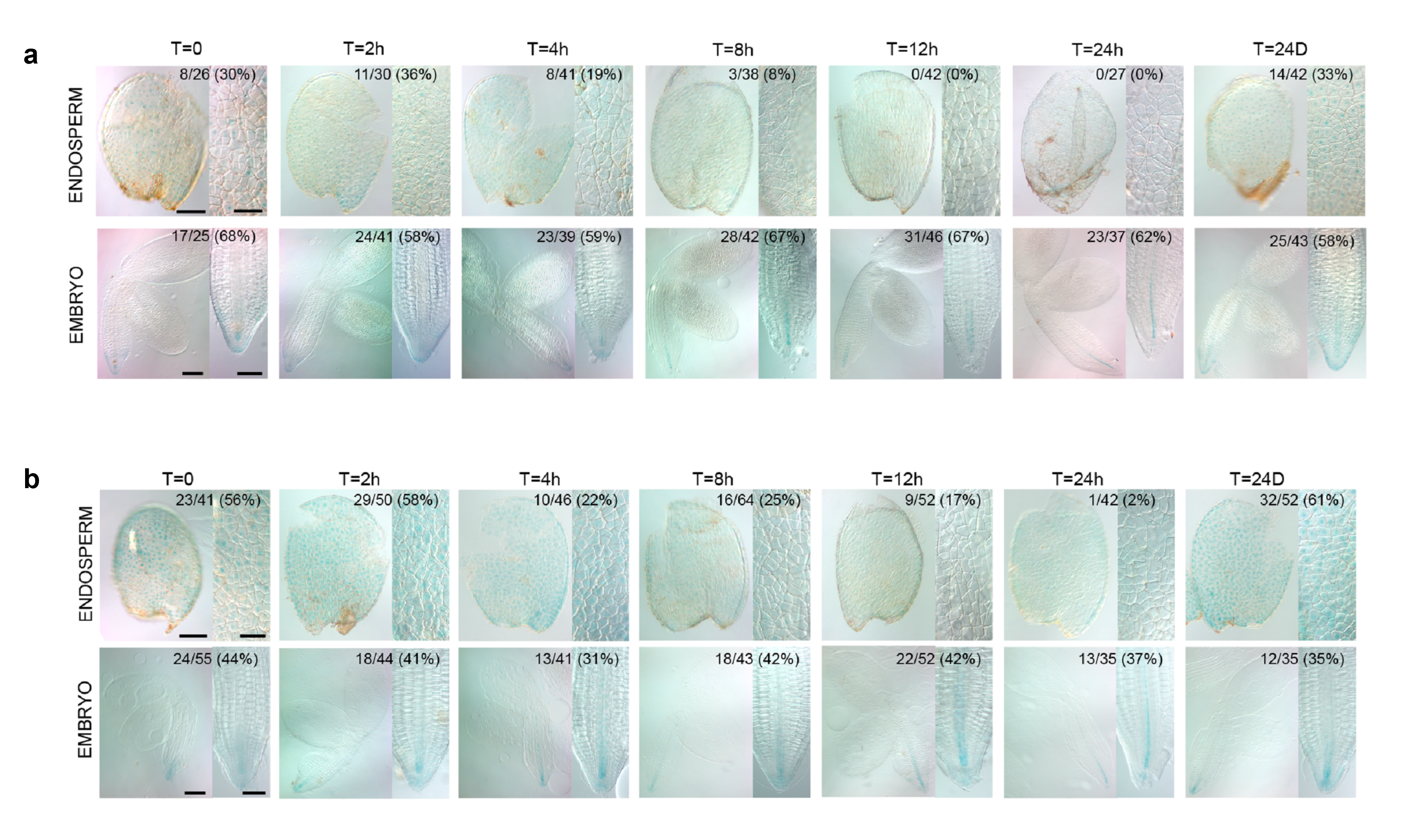

Supplement: Supplementary file 3 — Supplementary Information 3. [file 41598_2021_88874_MOESM3_ESM.tif]

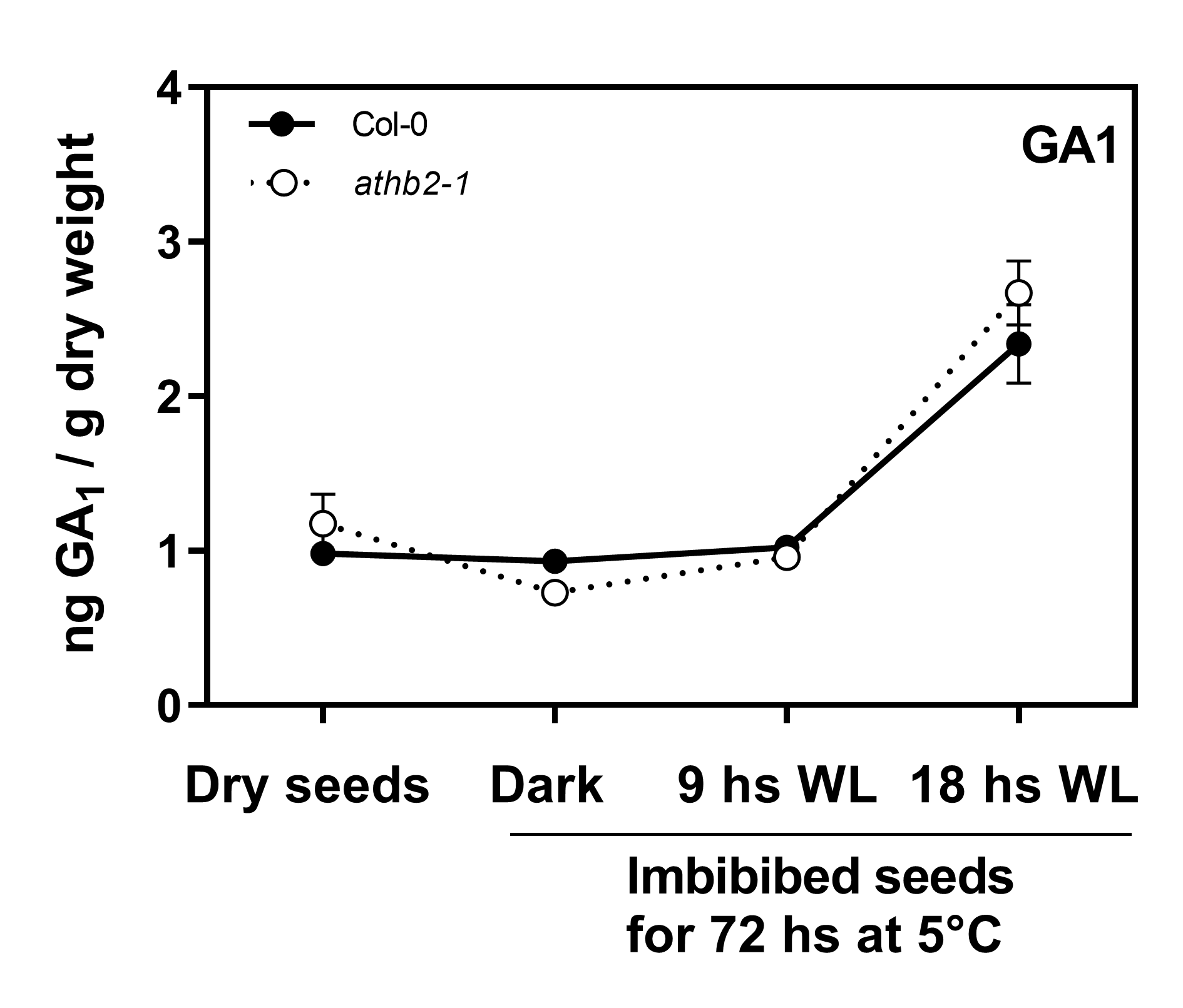

Supplement: Supplementary file 4 — Supplementary Information 4. [file 41598_2021_88874_MOESM4_ESM.tif]

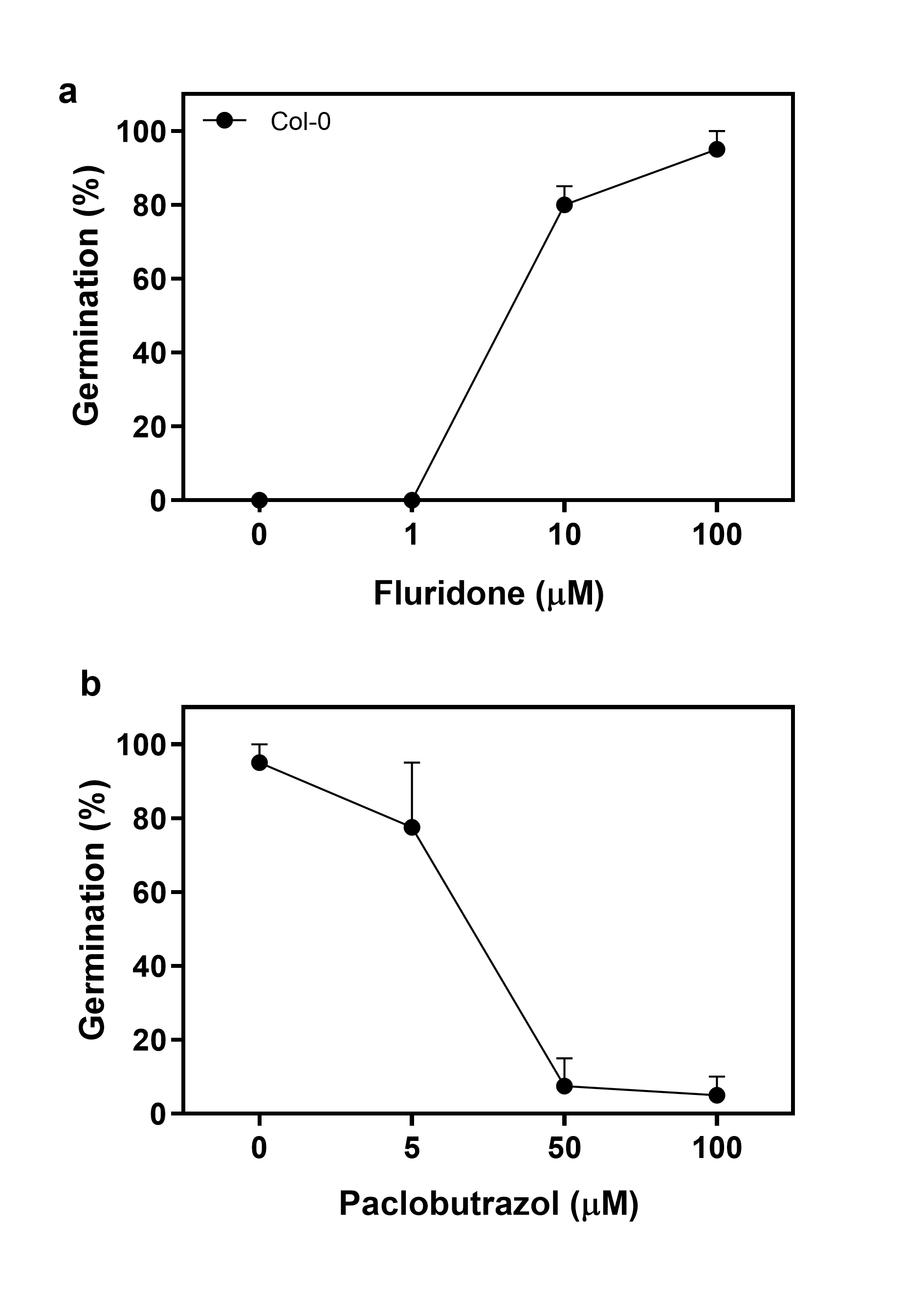

Supplement: Supplementary file 5 — Supplementary Information 5. [file 41598_2021_88874_MOESM5_ESM.tif]

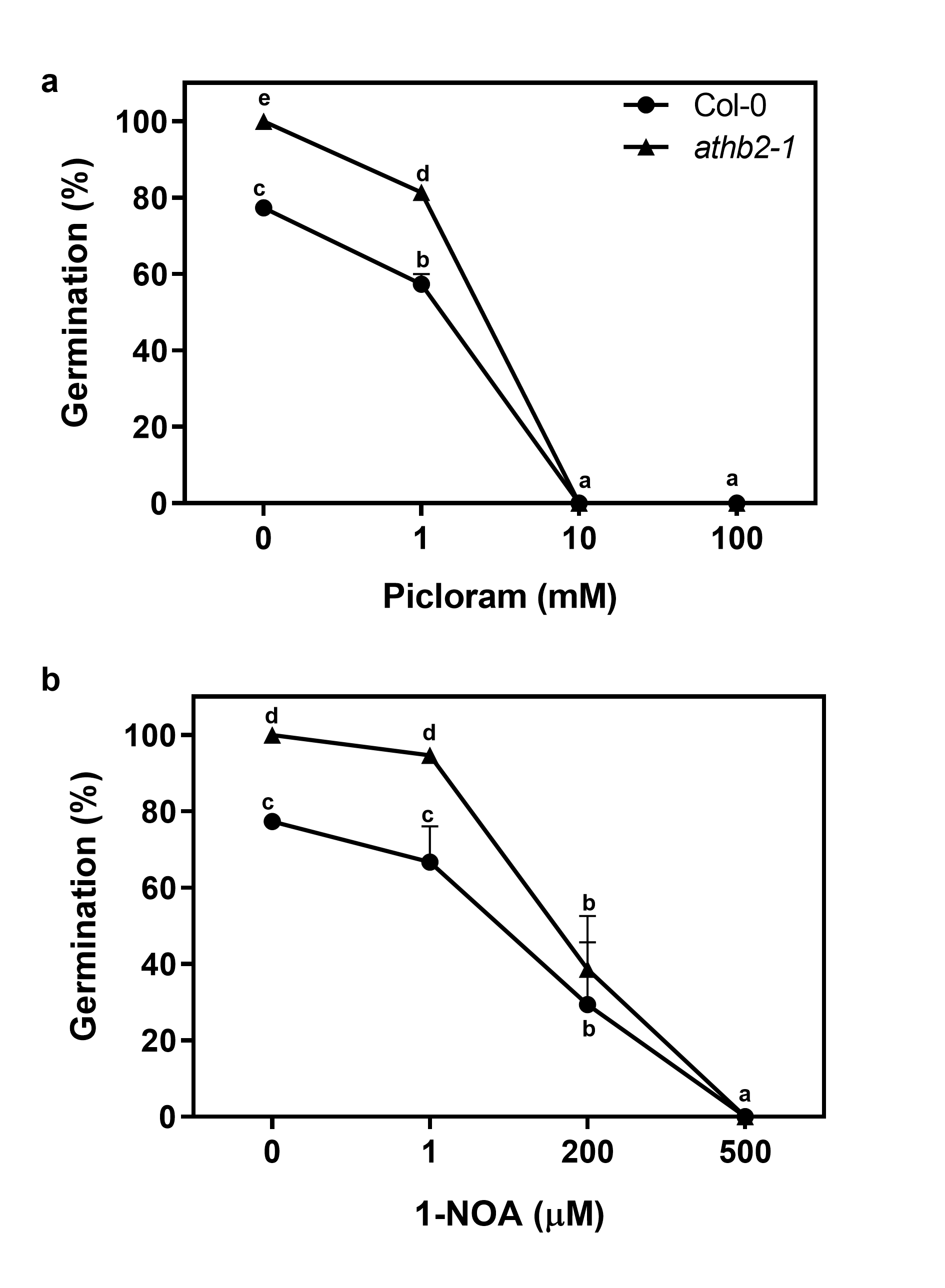

Supplement: Supplementary file 6 — Supplementary Information 6. [file 41598_2021_88874_MOESM6_ESM.tif]

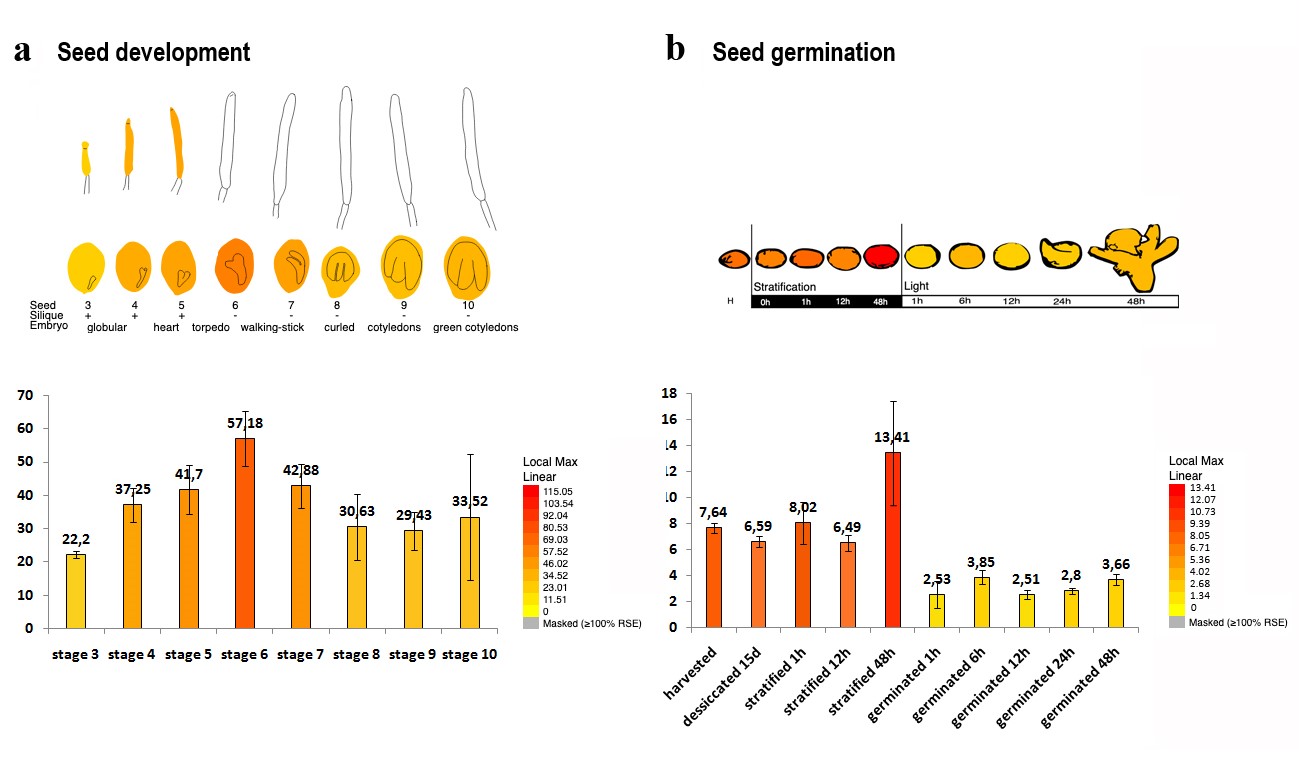

Supplement: Supplementary file 7 — Supplementary Information 7. [file 41598_2021_88874_MOESM7_ESM.jpg]
